# Comprehensive nursing in adenomyosis: a retrospective analysis of postoperative recovery, psychological status, and daily living ability

**DOI:** 10.3389/fpubh.2026.1861907

**Published:** 2026-06-17

**Authors:** Shuzhen Gan, Liu Yang

**Affiliations:** 1 Obstetrics Department, Ganzhou People’s Hospital, Ganzhou, China; 2 Reproductive Medicine Department, Ganzhou People’s Hospital, Ganzhou, China

**Keywords:** adenomyosis, comprehensive nursing, daily living ability, effect analysis, psychological status

## Abstract

**Objective:**

To explore the effect of comprehensive nursing on improving daily living ability in patients with adenomyosis through a retrospective analysis.

**Methods:**

A retrospective analysis was conducted on the data of 80 patients who underwent adenomyosis surgery at our hospital from October 2023 to March 2025. The patients were divided into a study group (comprehensive nursing) and a control group (conventional nursing) based on the nursing method, with 40 cases in each group. Propensity score matching was used to balance the baseline data of the two groups. The recovery indicators (postoperative exhaust time, fever time, first time to get out of bed, and hospital stay), Visual Analogue Scale (VAS) scores for pain, Hamilton Anxiety Scale (HAMA-l4) and Depression Scale (HAMD-24), Rosenberg Self-Esteem Scale, Quality of Life Scale, and Activities of Daily Living (ADL) Scale were compared between the two groups.

**Results:**

There was no significant difference in clinical data between the two groups (*p* > 0.05). The postoperative exhaust time, fever time, first time to get out of bed, and hospital stay in the study group were all shorter than those in the control group (*p* < 0.05). After nursing intervention, the VAS, HAMA-l4, and HAMD-24 scores in the study group were lower than those in the control group (*p* < 0.05), while the Rosenberg Self-Esteem Scale and ADL scores were higher than those in the control group (*p* < 0.05). All sub-scores of the Quality of Life Scale (physical function, mental function, cognitive level, social relationships) in the study group were higher than those in the control group (*p* < 0.05).

**Conclusion:**

Comprehensive nursing can promote rapid postoperative recovery, improve daily living ability, alleviate pain and psychological states, and enhance quality of life in patients with adenomyosis, providing a reference for clinical intervention.

## 1 Introduction

Adenomyosis, as a common gynecological disease among women of childbearing age, is characterized by the invasion of endometrial glands and stroma into the uterine myometrium, resulting in diffuse or localized lesions (1, 2). Epidemiological data indicate that the global prevalence of this disease in women of childbearing age is approximately 10–15%, with a high incidence in the 30–50 age group. About 50% of patients have concurrent uterine fibroids, forming a complex pathological state. This disease not only causes somatic symptoms such as menstrual disorders (increased menstrual flow, prolonged menstruation), progressive dysmenorrhea (pain score up to 7–9), and dyspareunia, but also triggers psychological problems such as anxiety and depression due to long-term chronic pain, impaired fertility, and restricted social functioning, forming a vicious cycle of “somatic-psychological-social function” (3–6). Traditional nursing models primarily focus on symptom control, such as pain management (providing only analgesics), menstrual care (guiding the use of sanitary products), and basic health education. However, this “passive response” model has significant shortcomings: first, it neglects psychosocial support, leading to social avoidance behaviors in patients due to disease-related shame and treatment uncertainty, resulting in a decline in Activities of Daily Living (ADL) scores; second, it lacks individualized nutritional guidance, with some patients exacerbating bleeding symptoms due to blind tonic intake (e.g., excessive consumption of red dates and brown sugar); third, it is insufficient in rehabilitation training, as postoperative patients may develop deep vein thrombosis (DVT) due to prolonged bed rest caused by fear of pain; fourth, family involvement is low, and inadequate family understanding of the disease results in ineffective care for patients, creating a gap in “hospital-family” nursing (7–9). In contrast, comprehensive nursing intervention, based on the “holistic person” theory, integrates the biopsychosocial medical model and employs multidimensional intervention strategies to improve patients’ daily living abilities (10). This study focuses on the improvement of daily living abilities (including somatic function, psychological function, and social function) in patients with adenomyosis. Through a controlled experimental design, it verifies the effectiveness of comprehensive nursing intervention in enhancing ADL scores, reducing complication rates, and shortening hospital stays. The expected outcomes will provide evidence-based support for clinical nursing practice, promote the transition of nursing models from “disease-centered” to “patient-centered,” and ultimately achieve the goal of “symptom relief-functional recovery-quality of life improvement” throughout the entire management process.

## 2 Materials and methods

### 2.1 Study data

This study conducted a retrospective analysis of patient data from individuals who underwent surgery for adenomyosis at our hospital between October 2023 and March 2025. After rigorous screening based on inclusion criteria, a total of 80 patients were enrolled in the study. The patients were divided into a study group receiving comprehensive nursing and a control group receiving conventional nursing, with 40 cases in each group. Propensity score matching was used to balance baseline data between the two groups. This study was approved by the Ethics Committee of our hospital and strictly adhered to the ethical guidelines outlined in the Declaration of Helsinki and relevant national medical data protection regulations. Given that this was a retrospective study with data sourced from existing clinical records and no additional risks imposed on patients, the Ethics Committee of our hospital waived the informed consent requirement. Privacy protection was ensured through measures such as anonymization of all case data and the establishment of a tiered access control mechanism.

### 2.2 Inclusion criteria

All participating patients met the diagnostic criteria for adenomyosis outlined in Obstetrics and Gynecology. The diagnosis was confirmed via transvaginal ultrasound or MRI and subsequently verified by postoperative pathological examination (11–13). All patients underwent total hysterectomy as their treatment. Patients were required to be aged l8 years or older, possess normal cognitive function, and have complete and detailed clinical records without any missing information.

This study excluded patients with severe comorbidities involving other vital organs, those with severe complications or acute critical conditions, and patients with psychiatric disorders or communication impairments. Additionally, patients with concurrent malignancies, severe cardiovascular or cerebrovascular diseases, or psychiatric disorders were excluded. Patients who developed severe postoperative complications, such as infection or hemorrhage, as well as those with poor compliance, were also excluded from this study.

### 2.3 Nursing methods

The control group received conventional nursing, which was delivered by staff nurses who had completed standard institutional training and held uniform clinical qualifications, primarily including basic nursing procedures, continuous monitoring of vital signs, and daily life interventions. Nurses provided detailed explanations to patients regarding the pathogenesis of adenomyosis, treatment objectives, and precautions to be taken during treatment. Prior to surgery, patients were instructed to fast for 12 h and abstain from drinking water for 4 h. Vital signs, including respiration, blood pressure, and heart rate, were closely monitored, and appropriate measures were taken immediately if any abnormalities were detected. Postoperatively, patients were allowed to resume a liquid diet only after the return of flatus, with a gradual transition to a regular diet based on their recovery progress (14, 15).

The study group employed a comprehensive nursing protocol that spanned the preoperative, intraoperative, and postoperative periods. In this study, the comprehensive nursing protocol is defined as a holistic, patient-centered care framework that integrates targeted clinical pathways and psychological support. The specific details are as follows: ① Preoperative nursing: During the nursing process, the frequency of patient rounds should be increased to guide patients in completing preoperative preparations while strengthening ward inspections. Nurses should instruct patients to perform regular vaginal care and cleaning and ensure strict disinfection of medical devices to prevent post-treatment infections. Prior to surgery, psychological counseling should be actively provided via a 30-min structured face-to-face education session to address potential adverse psychological states, alleviate patient anxiety, and foster treatment confidence, thereby ensuring active cooperation with clinical treatment and smooth progression of therapeutic procedures. ② Intraoperative nursing: During surgery, timely communication should be maintained with conscious patients to stabilize their emotions and prevent excessive fluctuations that could adversely affect the procedure. Personnel movement in the operating room should be controlled to minimize infection risks, while patient warmth (using forced-air warming blankets) and privacy should be prioritized. Nurses should maintain a composed mindset to effectively manage emergencies and assist the attending physician in completing the treatment. ③ Postoperative nursing: In the postoperative phase, pain levels should be promptly assessed using the Visual Analogue Scale (VAS), and effective pain relief measures implemented to prevent excessive pain from affecting treatment compliance. Postoperative precautions should be clearly communicated to stabilize patient emotions and reinforce treatment confidence, thereby supporting subsequent therapeutic efforts and alleviating pain to some extent. Nurses should intensify monitoring for potential complications such as deep vein thrombosis, constipation, and hemorrhage, minimizing risks through vigilant observation. Additionally, early rehabilitation nursing concepts can be integrated, combining dietary and exercise interventions (such as progressive early ambulation within 12 h post-surgery) to improve nutritional status and promote recovery (16, 17).

### 2.4 Observation indicators

#### 2.4.1 Recovery outcomes

Systematic recording and comparative analysis were conducted on postoperative exhaust time, fever duration, time to first ambulation, and hospital stay for both groups.

#### 2.4.2 Pain assessment

The Visual Analogue Scale (VAS) was used to evaluate pain levels before and after nursing intervention. The VAS scoring criteria were as follows: 0 points indicated no pain; 1–3 points represented mild pain; 4–6 points denoted moderate pain; and 7 points or higher signified severe pain.

#### 2.4.3 Psychological status

Psychological evaluation was performed using the Hamilton Anxiety Scale (HAMA-14) and Hamilton Depression Scale (HAMD-24) before nursing intervention (at admission) and after nursing intervention (at discharge). For HAMA-14: a total score <6 indicated normal; 6–14 suggested possible anxiety; 14–20 confirmed anxiety; 21–28 denoted marked anxiety; and >28 indicated severe anxiety. For HAMD-24: a total score <7 indicated normal; 7–17 suggested possible depression; 17–24 confirmed depression; and >24 indicated severe depression.

#### 2.4.4 Self-esteem

The Rosenberg Self-Esteem Scale was used for assessment. This scale consists of 10 items with a 4-point scoring system: “strongly disagree” (1 point), “disagree” (2 points), “agree” (3 points), and “strongly agree” (4 points). The total score ranges from 10 to 40, with higher scores indicating greater self-esteem.

#### 2.4.5 Activities of daily living (ADL)

Improvements in ADL scores were compared between the two groups before nursing intervention (at admission) and after nursing intervention (at discharge). The Barthel Index was used to calculate ADL scores, covering 14 activities such as dressing, bathing, telephoning, eating, shopping, and mobility. The total score ranges from 0 to 100, with higher scores reflecting stronger self-care abilities.

#### 2.4.6 Quality of life

The Quality of Life Scale was used to evaluate patients’ quality of life, encompassing four domains: physical function, mental function, cognitive level, and social relationships. Each domain was scored on a 100-point scale, with higher scores indicating better quality of life.

### 2.5 Data analysis

Images in this study were processed using GraphPad Prism 8. Data were organized and analyzed using SPSS 26.0. Continuous variables are presented as mean ± standard deviation (x ± s) or as median and interquartile range where appropriate.

Between-group comparisons for continuous variables were conducted using t-tests. Categorical variables are expressed as [*n* (%)], with between-group comparisons performed using chi-square (*X*^2^) tests. A *p*-value <0.05 was considered statistically significant.

## 3 Results

### 3.1 Clinical characteristics

The study group comprised 40 patients aged 20–52 years (mean age: 33.94 ± 6.24 years), with a body mass index (BMI) of 21–27 kg/m^2^ (mean: 24.11 ± 1.28 kg/m^2^) and 4–16 years of education (mean: 11.77 ± 2.38 years). The control group included 40 patients aged 20–52 years (mean age: 34.05 ± 6.82 years), with a BMI of 21–27 kg/m^2^ (mean: 23.99 ± 1.46 kg/m^2^) and 4–16 years of education (mean: 11.85 ± 2.46 years). No statistically significant differences in clinical characteristics were observed between the two groups (*p* > 0.05), indicating comparability (see Table 1).

**Table 1:** Comparison of clinical characteristics between the two groups.

		Research group	Control group	*t*	*p*
Number of cases	–	40	40	–	–
Age	–	20–52	20–52	–	–
	Mean	33.94 ± 6.24	34.05 ± 6.82	0.075	0.940
BMI	–	21–27	21–27	–	–
	Mean	24.11 ± 1.28	23.99 ± 1.46	0.391	0.697
Years of education	–	4–16	4–16	–	–
	Mean	11.77 + 2.38	11.85 + 2.46	0.148	0.883

BMI, body mass index; VAS, visual analogue scale; HAMA-14, 14-item hamilton anxiety scale; HAMD-24, 24-item hamilton depression scale; ADL, activities of daily.

### 3.2 Recovery outcomes

**Figure 1:**
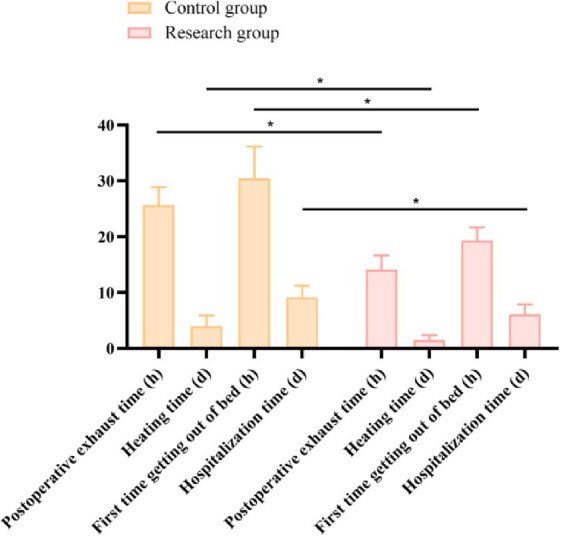
Comparison of recovery outcome indicators between the two groups. *Indicates a statistically significant difference between groups (*p* < 0.05).

The study group demonstrated significantly shorter postoperative exhaust time, fever duration, time to first ambulation, and hospital stay compared to the control group (14.12 ± 2.56/1.53 ± 0.85/19.32 ± 2.37/6.16 ± 1.72 vs. 25.67 ± 3.21/4.01 ± 1.89/30.46 ± 5.65/9. 17 ± 2.05; *p* < 0.05) (see Figure 1).

### 3.3 Pain assessment

**Figure 2:**
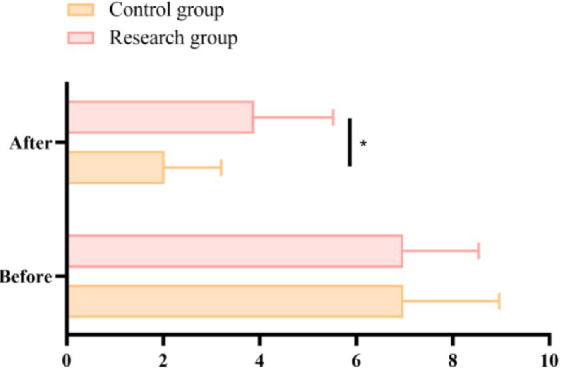
Comparison of VAS scores before and after intervention between the two groups. *Indicates a statistically significant difference between groups (*p* < 0.05).

No statistically significant difference in VAS scores was observed between the two groups before nursing intervention (*p* > 0.05). After intervention, the study group had significantly lower VAS scores than the control group (2.03 ± 1.17 vs. 3.88 ± 1.64; *p* < 0.05) (see Figure 2).

### 3.4 Psychological status

**Figure 3:**
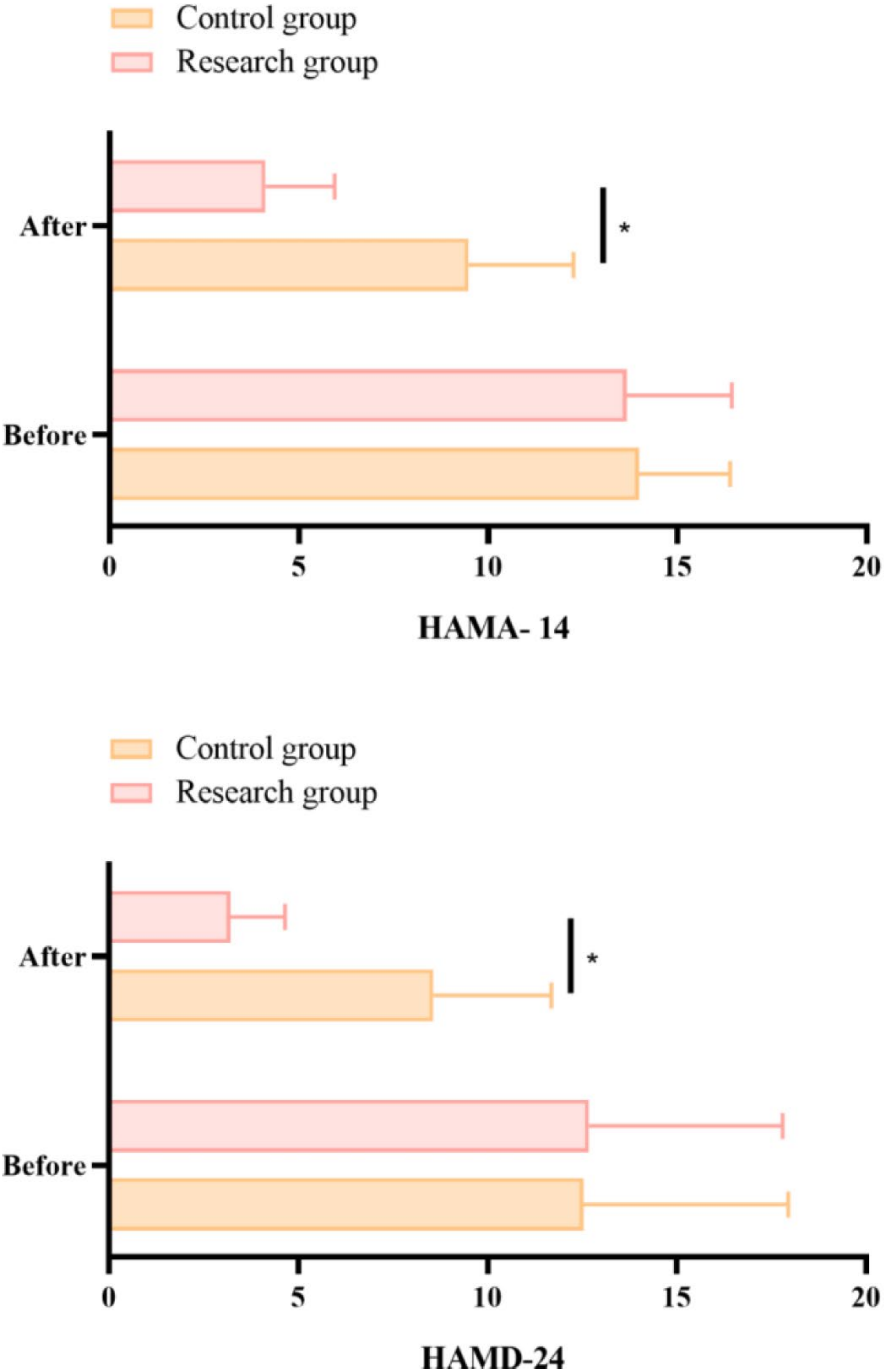
Comparison of HAMA-14 and HAMD-24 scores before and after intervention between the two groups. *Indicates a statistically significant difference between groups (*p* < 0.05).

No statistically significant differences in HAMA-14 and HAMD-24 scores were observed between the two groups before nursing intervention (*p* > 0.05). After intervention, the study group had significantly lower HAMA-14 and HAMD-24 scores than the control group (4.12 ± 1.82/3.21 ± 1.44 vs. 9.48 ± 2.77/8.57 ± 3. 11; *p* < 0.05) (see Figure 3).

### 3.5 Self-esteem

**Figure 4:**
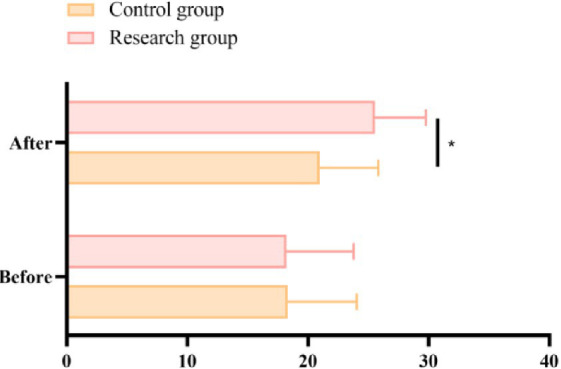
Comparison of rosenberg scores before and after intervention between the two groups. *Indicates a statistically significant difference between groups (*p* < 0.05).

No statistically significant difference in Rosenberg Self-Esteem Scale scores was observed between the two groups before nursing intervention (*p* > 0.05). After intervention, the study group had significantly higher Rosenberg scores than the control group (25.56 ± 4. 19 vs. 20.98 ± 4.85; *p* < 0.05) (see Figure 4).

### 3.6 Activities of daily living (ADL)

**Figure 5:**
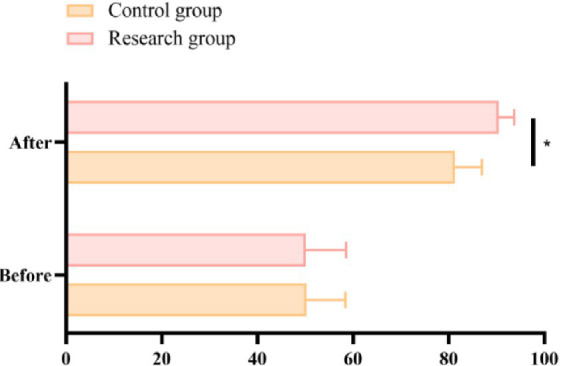
Comparison of ADL scores before and after intervention between the two groups. *Indicates a statistically significant difference between groups (*p* < 0.05).

No statistically significant difference in ADL scores was observed between the two groups before nursing intervention (*p* > 0.05). After intervention, the study group had significantly higher ADL scores than the control group (90.45 ± 3.22 vs. 81.27 ± 5.67; *p* < 0.05) (see Figure 5).

### 3.7 Quality of life

**Figure 6:**
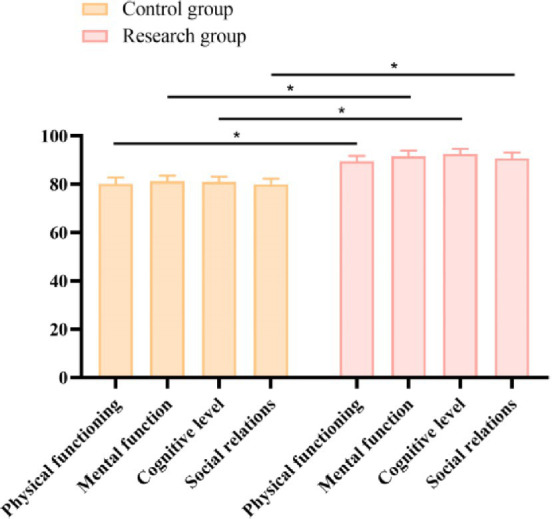
Comparison of quality of life scale scores between the two groups. *Indicates a statistically significant difference between groups (*p* < 0.05).

The study group demonstrated significantly higher scores across all domains of the Quality of Life Scale (physical function, mental function, cognitive level, social relationships) compared to the control group (89.56 ± 2.11/91.58 ± 2.37/92.44 ± 2.18/90.73 ± 2.36 vs. 80.17 ± 2.65/81.18 ± 2.33/80.94 ± 2.17/79.96 ± 2.29; *p* < 0.05) (see Figure 6).

## 4 Discussion

Currently, the pathogenesis of adenomyosis remains incompletely understood, but mainstream theories suggest a strong association with endometrial basal layer injury, hormonal imbalances, and immune dysregulation. A history of intrauterine procedures (e.g., induced abortion, cesarean section) disrupts the integrity of the endometrial-myometrial interface, providing a pathological basis for ectopic glandular implantation. Estrogen exacerbates lesion progression by promoting endometrial proliferation and inflammatory cytokine release, while progesterone resistance prevents normal shedding of ectopic endometrium, leading to persistent inflammatory responses (18, 19). This pathological process results in uterine enlargement, increased tissue hardness, and impaired uterine contractility, subsequently causing menorrhagia (average blood loss increased by 2–3 times compared to normal) and severe anemia (hemoglobin levels as low as 60–80 g/L). In clinical practice, hysterectomy offers definitive treatment but involves invasive procedures that prolong postoperative recovery and may lead to long-term complications such as pelvic floor dysfunction and sexual dysfunction (20–22). For patients wishing to preserve fertility, pharmacological therapies (e.g., GnRH-a, levonorgestrel-releasing intrauterine system) can alleviate symptoms but require long-term management and are associated with side effects like hypoestrogenism and breakthrough bleeding (23). Therefore, improving patients’ activities of daily living through nursing interventions has become critical for enhancing treatment adherence and quality of life.

First, the study group demonstrated significantly shorter postoperative exhaust time, fever duration, time to first ambulation, and hospital stay compared to the control group, highlighting the central role of comprehensive nursing in promoting rapid postoperative recovery. Through multidimensional interventions—including preoperative vaginal care and hygiene enhancement, strict control of personnel movement and thermal insulation during surgery, and postoperative early mobilization guidance with complication prevention—comprehensive nursing effectively optimized the physiological recovery pathway. Standardized preoperative vaginal care reduced postoperative infection risks, creating favorable conditions for early recovery.

Intraoperative measures, such as limiting surgical personnel movement and implementing thermal insulation, minimized stress responses induced by environmental factors, thereby reducing complications like postoperative fever. Postoperative early ambulation guidance and rehabilitation training accelerated gastrointestinal functional recovery, shortened exhaust time, and prevented complications such as deep vein thrombosis, further reducing hospital stays. This multi-stage synergistic rehabilitation mechanism demonstrated significant advantages in shortening postoperative recovery time, enabling faster rehabilitation and lower medical costs for patients, consistent with findings from previous similar studies.

Second, the study group exhibited significantly lower VAS scores than the control group after nursing intervention, indicating the unique advantages of comprehensive nursing in pain management. Comprehensive nursing employed a multimodal analgesia strategy combining pharmacological pain relief with psychological counseling, effectively reducing patients’ pain perception. Preoperative psychological counseling alleviated patient anxiety, promoting a more stable mental state during treatment and reducing pain sensitivity caused by tension. Intraoperative emotional stabilization and optimized surgical environment minimized the impact of surgical stress on pain. Postoperative timely pain assessment and targeted interventions, such as pharmacological analgesia and physical therapy, effectively controlled pain levels. This holistic pain management mechanism not only reduced physical suffering but also decreased psychological stress induced by pain, creating a virtuous cycle that facilitated overall patient recovery.

Third, the study group demonstrated significantly lower HAMA-l4 and HAMD-24 scores compared to the control group, revealing the notable effectiveness of comprehensive nursing in improving patients’ psychological states. Comprehensive nursing established a complete psychological intervention system through preoperative psychological counseling, intraoperative emotional stabilization, and postoperative sustained psychological support. Preoperative targeted counseling addressed potential adverse psychological states in patients, helping them build treatment confidence and alleviate anxiety caused by insufficient disease awareness.

Intraoperative timely communication with conscious patients stabilized their emotions, avoiding adverse effects of emotional fluctuations on the surgical process. Postoperative clarification of precautions and sustained psychological support helped patients develop a positive treatment mindset, effectively relieving postoperative anxiety and depressive moods. This psychological intervention mechanism improved patients’ psychological states, not only enhancing treatment adherence but also accelerating physiological recovery, forming a psychophysiological synergistic rehabilitation model (24, 25).

Moreover, the study group scored higher on the Rosenberg Self-Esteem Scale than the control group, indicating the positive role of comprehensive nursing in elevating patients’ self-esteem levels. Through multidimensional interventions, comprehensive nursing helped patients rebuild positive self-perception and sense of value. Preoperative detailed explanations of disease mechanisms and treatment goals enhanced patients’ sense of control over the disease and improved their self-efficacy. Intraoperative respect for patient privacy and protection of dignity maintained their self-worth identification. Postoperative positive feedback on rehabilitation progress and the establishment of social support networks reinforced patients’ self-affirmation and value experience. This elevation in self-esteem not only improved patients’ psychological states but also strengthened their motivation for social functional recovery, providing robust psychological support for their return to normal life.

Additionally, the study group exhibited higher ADL scores than the control group, reflecting the significant effectiveness of comprehensive nursing in enhancing patients’ activities of daily living. Comprehensive nursing comprehensively promoted the recovery of patients’ daily living abilities through early rehabilitation training, social support network construction, and life skill guidance. The integration of early postoperative rehabilitation training accelerated the recovery of patients’ physical functions, improving basic life skills such as dressing, bathing, and eating. The establishment of social support networks, including family involvement in care and peer support groups, enhanced patients’ sense of social belonging and support, facilitating social functional recovery. Specific guidance on life skills, such as dietary transitions and activity training, helped patients gradually rebuild independent living abilities. This all-encompassing mechanism for promoting daily living abilities enabled patients to achieve rapid recovery not only in physiological indicators but also in a qualitative improvement in daily living abilities, laying a solid foundation for their reintegration into social life (26–28).

Finally, the study group demonstrated higher scores across all domains of the quality of life scale compared to the control group, indicating the comprehensive advantages of comprehensive nursing in holistically improving patients’ quality of life. Through synergistic promotion across four dimensions—physical function, mental function, cognitive level, and social relationships—comprehensive nursing achieved an overall enhancement in patients’ quality of life. Rapid recovery of physical function provided a solid physiological foundation for patients, improved mental function strengthened psychological resilience, elevated cognitive levels enhanced patients’ correct understanding of the disease and treatment, and optimized social relationships reinforced their social support networks. This multidimensional mechanism for improving quality of life enabled patients to achieve not only physiological recovery but also comprehensive growth in psychological, social, and cognitive aspects during treatment, ultimately realizing a holistic improvement in quality of life and providing strong support for long-term health management (29, 30). These positive outcomes are highly consistent with findings from recent literature focusing on structured clinical paths and psychological support in gynecological care. For instance, Feng et al. demonstrated that comprehensive nursing interventions significantly alleviate negative emotions, reduce postoperative pain, and accelerate physical recovery in patients undergoing gynecological surgeries (31). Crucially, evidence from group-based trajectory modeling highlights that reproductive-age women undergoing benign hysterectomy frequently require extended postoperative prescriptions for antidepressant and antianxiety medications, revealing a persistent long-term psychological burden that demands early clinical attention (32). To address these challenges, the clinical application of comprehensive nursing has been increasingly optimized, demonstrating a profound capacity to streamline surgical paths, significantly reduce operative stress, and minimize intraoperative complications in women with benign uterine disorders (33). By comparing our work with these established findings, the unique clinical value of a comprehensive nursing protocol for adenomyosis patients becomes even more apparent. The implementation of holistic, targeted interventions successfully bridges the gap between purely clinical treatments and patients’ psychological resilience, ultimately leading to a more profound improvement in their daily living abilities and overall long-term wellness.

While this study validated the role of comprehensive nursing in improving daily living abilities among patients with adenomyosis, several limitations warrant objective consideration. First, as a single-center retrospective study, the data source was confined to a single medical institution, potentially introducing selection bias and requiring caution in generalizing the conclusions. Importantly, the retrospective nature of this design may inherently affect the consistency of historical clinical data collection, as some detailed behavioral or psychological nuances might not have been uniformly documented in standard medical records. Second, the relatively small sample size, despite balancing baseline characteristics through propensity score matching, suggests that larger multicenter studies could further enhance the statistical power and clinical generalizability of the results. In the future, well-designed prospective, multi-center studies with expanded cohorts are strongly warranted to cross-validate our findings and evaluate the long-term sustainability of the comprehensive care model. Third, the follow-up period covered only the short-term postoperative recovery phase, lacking long-term quality of life tracking, such as changes in ADL scores at 6 months or 1 year postoperatively, persistence of chronic pain, and social functional rehabilitation outcomes, limiting a comprehensive evaluation of the long-term benefits of comprehensive nursing. Additionally, the study did not incorporate qualitative data on patients’ subjective experiences, such as acceptance of nursing measures and psychological adaptation processes; future research could integrate quantitative and qualitative methods to provide a more holistic depiction of the actual effects of nursing interventions.

Looking ahead, research could be expanded and deepened across multiple dimensions. Methodologically, prospective randomized controlled trials could be conducted to reduce confounding factors and enhance the reliability of causal inferences through stricter randomization and blinding designs. In terms of intervention content, the application of digital nursing tools could be explored, such as using wearable devices to monitor patients’ vital signs and activity levels in real time, combined with artificial intelligence algorithms to predict complication risks and enable dynamic adjustments to personalized nursing plans. For outcome assessment, follow-up could be extended to 1–2 years postoperatively, focusing on observing social functional rehabilitation, occupational resumption, and long-term psychological state changes to comprehensively evaluate the impact of comprehensive nursing on patients’ holistic health. Mechanistically, the neuroendocrine mechanisms underlying the improvement in daily living abilities by comprehensive nursing could be further elucidated, such as through biomarker detection of salivary cortisol and serum brain-derived neurotrophic factor to reveal the intrinsic links between psychological interventions and physiological recovery. By exploring these directions, comprehensive nursing models could advance from empirical summaries toward precision and intelligence, ultimately achieving a transformative leap from “postoperative rapid recovery” to “whole-lifecycle health management.”

## 5 Conclusion

This study systematically validated the role of comprehensive nursing in improving daily living abilities among patients with adenomyosis through a retrospective analysis of data from 80 surgical cases. The results demonstrated that compared to conventional nursing, comprehensive nursing significantly shortened postoperative exhaust time, fever duration, time to first ambulation, and hospital stay; effectively alleviated pain perception; improved psychological states such as anxiety and depression; elevated self-esteem levels; and enhanced quality of life. Specifically, comprehensive nursing formed a “physiological-psychological-social” three-dimensional rehabilitation promotion mechanism through multistage coordinated interventions, including preoperative enhanced vaginal care and psychological counseling, intraoperative infection risk control and thermal insulation, and postoperative early rehabilitation training and complication prevention. This mechanism not only accelerated patients’ physical functional recovery but also achieved qualitative improvements in daily living abilities through psychological support and social functional rehabilitation. Despite limitations such as its single-center retrospective design, limited sample size, and short follow-up period, the study’s conclusions provide important references for clinical nursing practice: the comprehensive nursing model effectively addresses the limitations of traditional nursing’s singularity and enables a true transition from “disease treatment” to “holistic health” nursing through personalized, whole-cycle intervention strategies, demonstrating significant clinical value and application prospects for optimizing postoperative management and improving long-term quality of life in patients with adenomyosis.

## Data Availability

The original contributions presented in the study are included in the article/supplementary material, further inquiries can be directed to the corresponding author.
